# Supplementary figures and images for: Recruitment of Scc2/4 to double-strand breaks depends on γH2A and DNA end resection
Source: Life Sci Alliance. 2022 Jan 27;5(5):e202101244. doi: 10.26508/lsa.202101244 (PMC8807874; doi:10.26508/lsa.202101244)

Full Blot Fig 3B

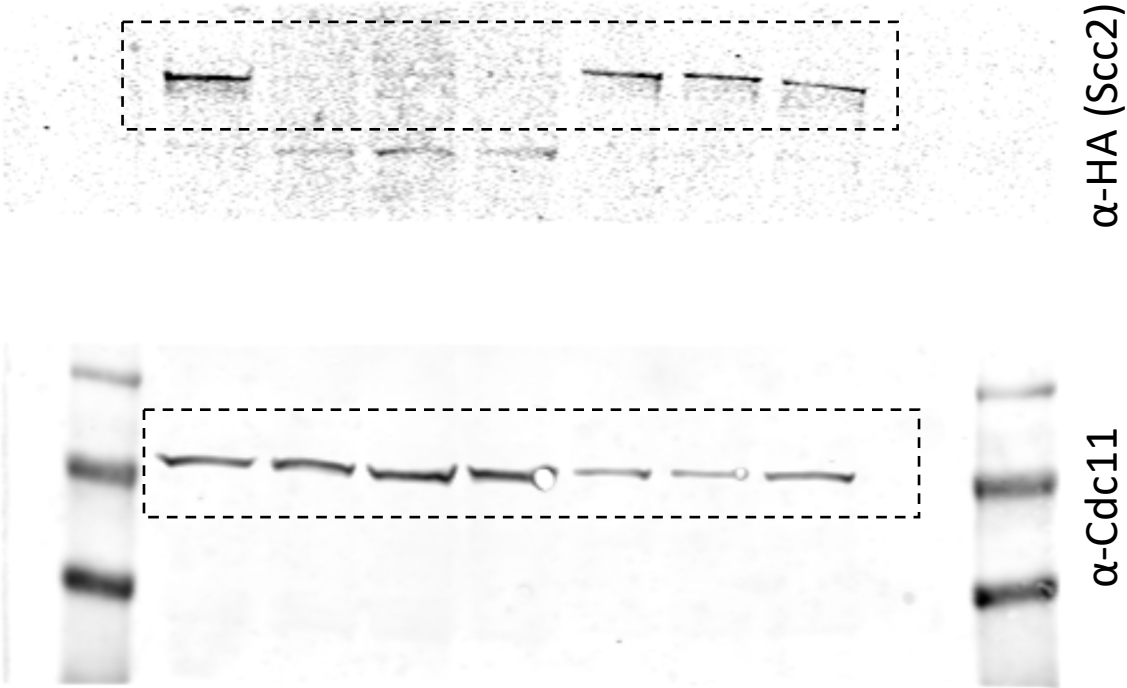

Supplement: Supplementary file 1 [file LSA-2021-01244_SdataF3.pdf]

Full Blot Fig 4E

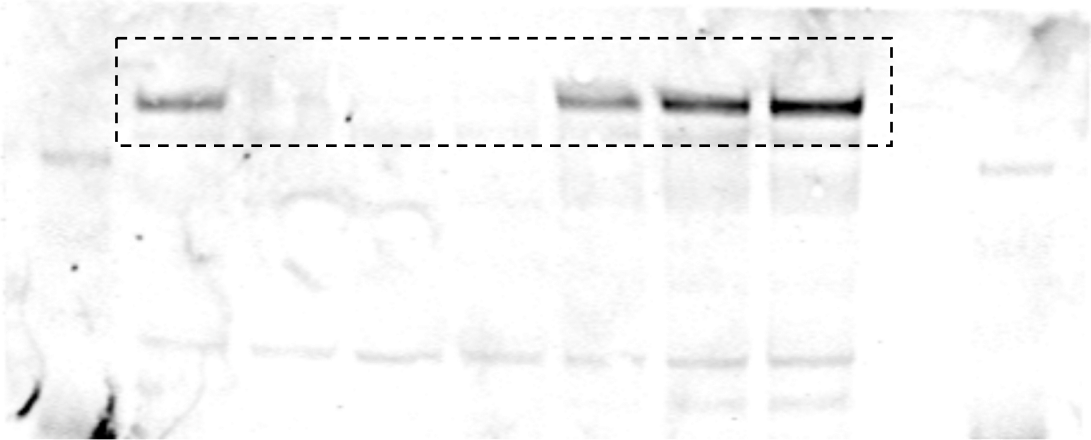

α-HA (Scc1)

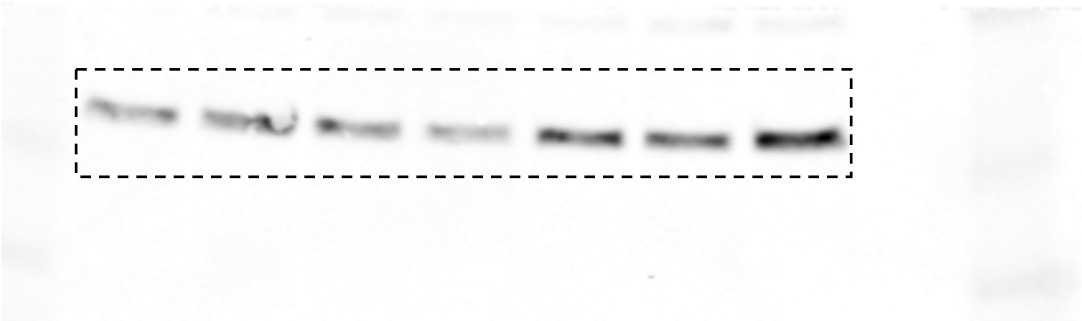

α-H3

Supplement: Supplementary file 2 [file LSA-2021-01244_SdataF4.pdf]

Full Blot Fig S4A

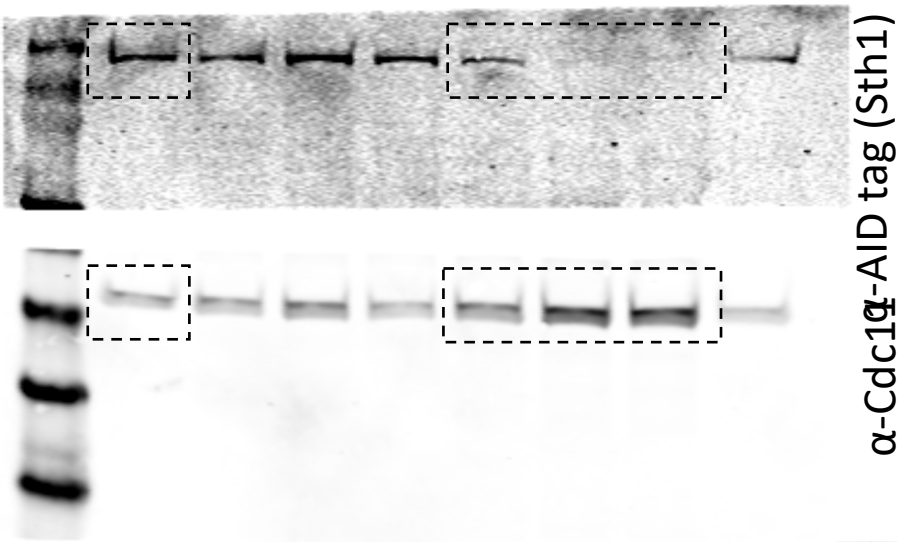

Southern Blot S4B

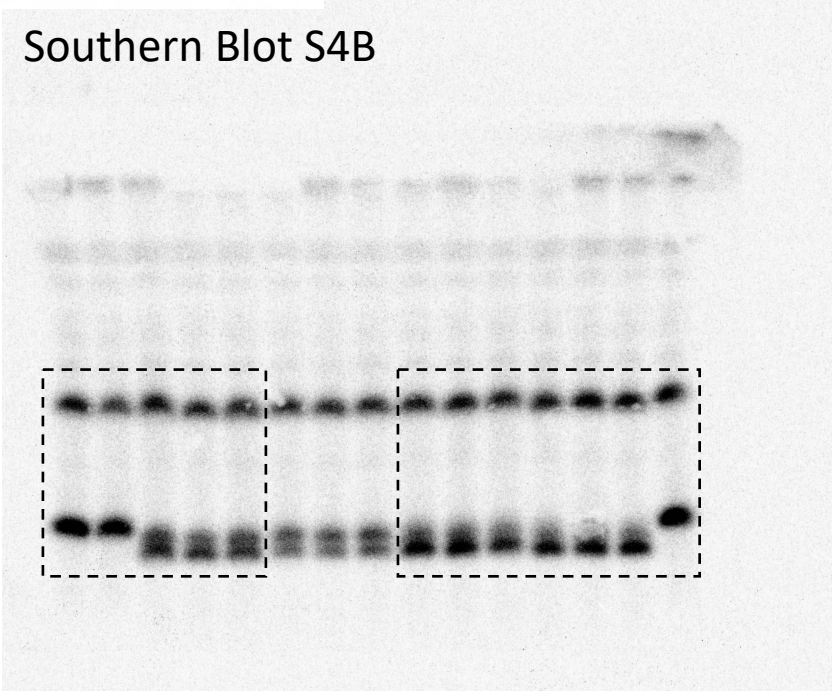

Pulsed-field Gel S4C

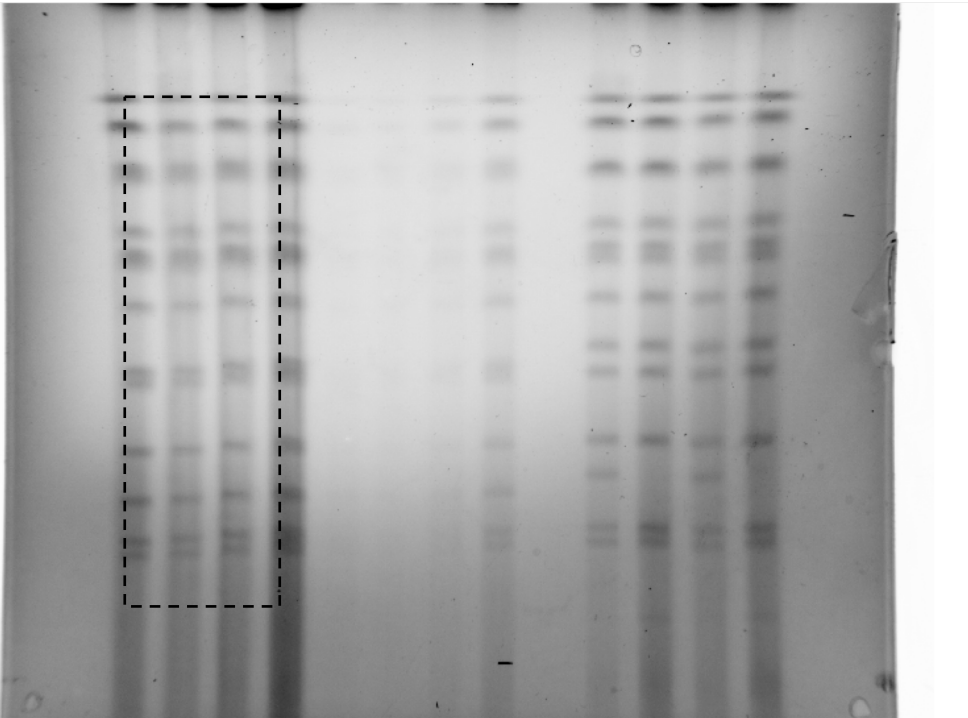

Supplement: Supplementary file 3 [file LSA-2021-01244_SdataFS4.pdf]
